# DNA methylation landscape of the genes regulating D-serine and D-aspartate metabolism in post-mortem brain from controls and subjects with schizophrenia

**DOI:** 10.1038/s41598-018-28332-x

**Published:** 2018-07-05

**Authors:** Simona Keller, Daniela Punzo, Mariella Cuomo, Ornella Affinito, Lorena Coretti, Silvia Sacchi, Ermanno Florio, Francesca Lembo, Massimo Carella, Massimiliano Copetti, Sergio Cocozza, Darrick T. Balu, Francesco Errico, Alessandro Usiello, Lorenzo Chiariotti

**Affiliations:** 1 Department of Molecular Medicine and Medical Biotechnology, University of Naples “Federico II”, 80131 Naples, Italy; 2 Endocrinology and Molecular Oncology Institute (I.E.O.S.), National Research Council (C.N.R.), 80131 Naples, Italy; 3 Laboratory of Behavioural Neuroscience, Ceinge Biotecnologie Avanzate, 80145 Naples, Italy; 4 Department of Environmental, Biological and Pharmaceutical Sciences and Technologies, Università degli Studi della Campania “Luigi Vanvitelli”, 81100 Caserta, Italy; 5 Dipartimento di Biotecnologie e Scienze della Vita, Università degli Studi dell’Insubria, 21100 Varese, Italy; 6 The Protein Factory, Politecnico di Milano and Università degli studi dell’Insubria, 20131 Milano, Italy; 7 Department of Medicine, University of California, San Diego UCSD, La Jolla, 95000 CA USA; 8 Medical Genetics Unit, IRCCS Casa Sollievo della Sofferenza, 71013 San Giovanni Rotondo, FG Italy; 9 Unit of Biostatistics, IRCCS Casa Sollievo della Sofferenza, 71013 San Giovanni Rotondo, FG Italy; 10 Department of Psychiatry, Harvard Medical School, Boston, 02115 MA USA; 11 Translational Psychiatry Laboratory, McLean Hospital, Belmont, 02478 MA USA; 12 Department of Agricultural Sciences, University of Naples “Federico II”, 80055 Portici, Italy

## Abstract

The spatio-temporal regulation of genes involved in the synthesis and degradation of D-serine and D-aspartate such as *serine racemase* (*SR*), *D-amino acid oxidase* (*DAO*), *G72* and *D-aspartate oxidase* (*DDO*), play pivotal roles in determining the correct levels of these D-amino acids in the human brain. Here we provide a comprehensive analysis of mRNA expression and DNA methylation status of these genes in post-mortem samples from hippocampus, dorsolateral prefrontal cortex, and cerebellum from patients with schizophrenia and non-psychiatric controls. DNA methylation analysis was performed at an ultradeep level, measuring individual epialleles frequency by single molecule approach. Differential CpG methylation and expression was detected across different brain regions, although no significant correlations were found with diagnosis. *G72* showed the highest CpG and non-CpG methylation degree, which may explain the repression of *G72* transcription in the brain regions considered here. Conversely, in line with the sustained *SR* mRNA expression in the analyzed areas, very low methylation levels were detected at this gene’s regulatory regions. Furthermore, for *DAO* and *DDO*, our single-molecule methylation approach demonstrated that analysis of epiallele distribution was able to detect differences in DNA methylation representing area-specific methylation signatures, which are likely not detectable with targeted or genome-wide classic methylation analyses.

## Introduction

Free D-serine (D-Ser) and D-aspartate (D-Asp) act as a co-agonist and agonist at *N*-methyl-D-aspartate receptors (NMDARs), respectively, and influence numerous brain functions dependent by this subclass of glutamate receptors^1–4^. These D-amino acids are present in the mammalian brain with an age- and region-specific distribution pattern^5–7^. Based on the NMDAR hypofunction hypothesis of schizophrenia^8,9^, perturbation of D-Ser and D-Asp metabolism has been implicated as a contributor to the pathogenesis of this devastating mental illness^10,11^. In this regard, altered serum and CSF levels of D-Ser have been observed in patients affected by schizophrenia, compared to controls^12–16^. Similarly, a significant decrease in D-Asp levels in the prefrontal cortex has been also recently reported in two different brain cohorts of post-mortem schizophrenia patients^17,18^. D-Ser and D-Asp levels are regulated by different enzymes, whose genetic polymorphisms and altered expression levels have been associated with schizophrenia^14,19–24^. While in mammals the synthesizing enzyme for D-Asp is still questioned, it is well established that D-aspartate oxidase (DDO) is the only known enzyme able to degrade endogenous D-Asp^25^. On the other hand, serine racemase (SR) and D-amino acid oxidase (DAO) are involved in D-Ser synthesis and degradation, respectively^26,27^. Interestingly, reduction of DAO activity, through the increase of endogenous D-Ser levels, has been suggested as a pharmacological tool for ameliorating schizophrenia symptoms. In particular, add-on therapy with a DAO inhibitor, sodium benzoate, improves schizophrenia symptomatology, even in clozapine-resistant patients^28,29^. Another protein, pLG72, also called D-amino acid oxidase activator (DAOA), encoded by the *G72* gene, has been proposed to function as a DAO modulator. However, it is currently debated if this gene is actually expressed in the brain. Interestingly, pLG72 has been found in the human plasma and identified as a potential biomarker for schizophrenia since its blood levels are distinctively higher in patients with schizophrenia than in healthy individuals^30^. It is also still unclear how pLG72 regulates DAO activity^31^. Indeed, independent findings demonstrate that pLG72 could either increase^20^ or decrease^32,33^ the activity of DAO.

As temporal and regional D-Asp and D-Ser levels are thought to play a role in NMDAR-related synaptic plasticity, morphology and functioning^11,34^, it is likely that the expression program of the genes regulating the metabolism of these D-amino acids is strictly orchestrated in the mammalian brain. Accordingly, we hypothesized that epigenetic mechanisms could drive the appropriate spatio-temporal modulation of these genes in human brain. Therefore, it could be envisaged that altered epigenetic profiles at these genes may be associated with human brain disorders, including schizophrenia. In particular, alteration of DNA methylation patterns in brain at CpG sites has been postulated as a factor contributing to the pathophysiology of mental disorders^35–40^. Recent studies have also reported that non-CpG methylation (mCH) is present at significant levels in brain cells and may have a functional role^41–46^. However, to date very few studies have addressed the role of non-CpG methylation profiles in human brain disorders.

In this work, we performed an ultra-deep analysis of DNA methylation, at CpG and non-CpG sites, as well as expression profiles of all known D-Ser and D-Asp modulating genes (*SR*, *DAO*, G72 and *DDO*) in several brain areas of controls and subjects with schizophrenia. In addition to conventional average-based DNA methylation analysis, we also applied a single molecule methylation approach (epiallele classes and distribution analyses) in order to finely map cell to cell methylation differences in specific brain areas and to evaluate whether these ultra-deep methylation profiles may distinguish brain areas within each individual and/or be associated to schizophrenia diagnosis.

## Results

### DNA methylation and mRNA expression of the *DAO* gene in non-psychiatric subjects and patients with schizophrenia

**Figure 1:**
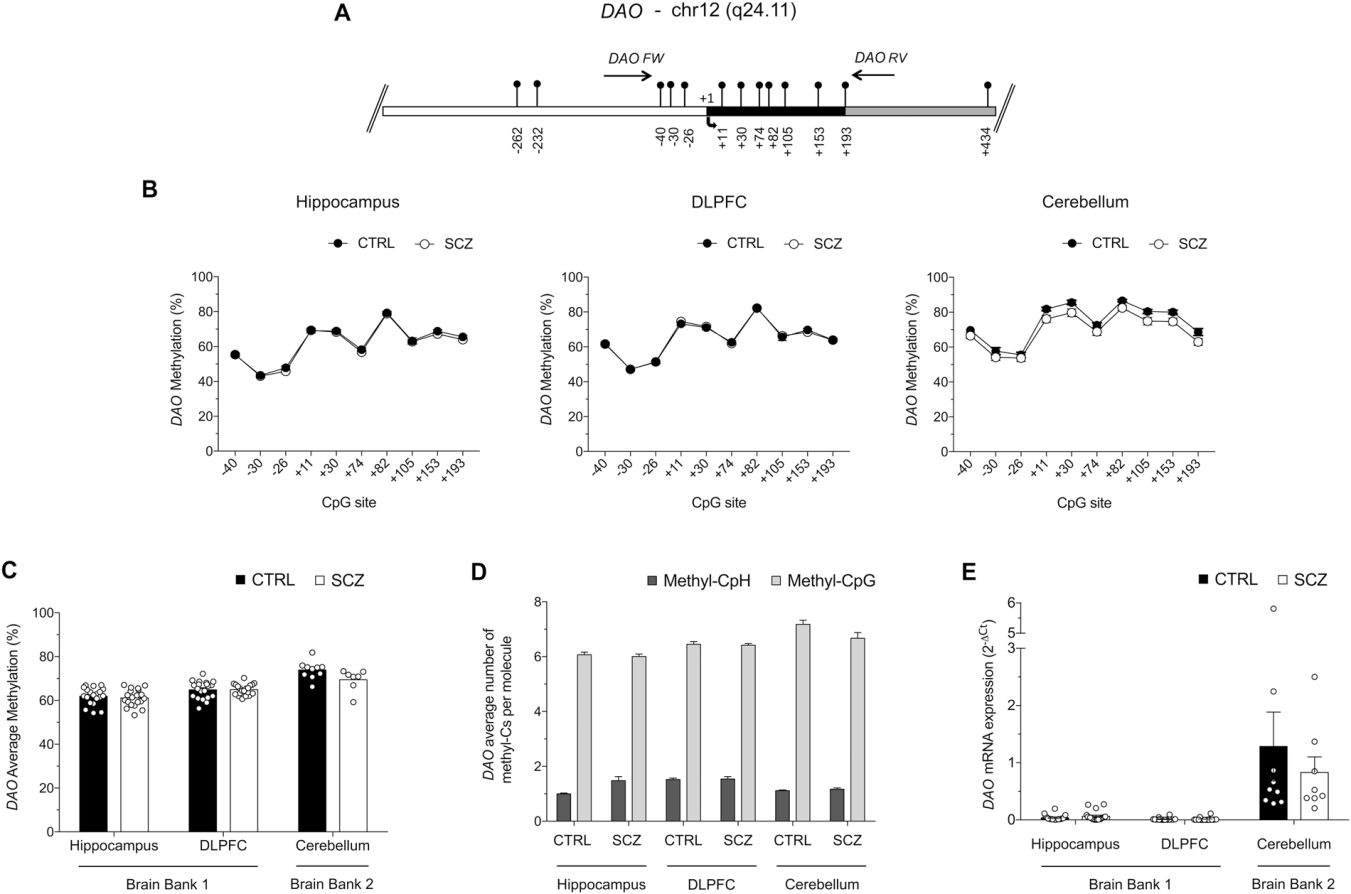
Epigenetic analysis of *DAO*. (**A**) Structure of the *DAO* gene showing the putative regulatory upstream region (white box), exons (black box) and first intron (grey box). Transcriptional start site (+1) is indicated by an arrow. Vertical bars represent the relative position of each CpG site and the arrows indicate the primer positions. The gene sequence was retrieved by Ensemble database accession number: DAO ENSG00000110887. (**B**) The methylation average at each CpG site, between the two groups (CTRL: black circles; SCZ; open circles) for each region (HIPP, DLPFC and CB) is reported in the graphs. (**C**) The average methylation of controls (CTRL) and subjects with schizophrenia (SCZ) in hippocampus (HIPP), dorsolateral-prefrontal cortex (DLPFC) and cerebellum (CB) areas is reported in the histogram. (**D**) For each sample group and brain area, the number of mCpH and mCpG per molecule was calculated as described in the Materials and Methods section. The percent values of non-CpG methylation are reported in Table S2. (**E**) DAO mRNA expression levels in the HIPP, DLPFC and CB of control and schizophrenia patients were detected by quantitative RT-PCR and expressed as 2^−ΔCt^. All data are shown as the mean ± standard error (SEM).

We first examined the methylation status of the gene encoding the D-Ser degrading enzyme, DAO, in the hippocampus (HIPP) and dorsolateral prefrontal cortex (DLPFC) from non-psychiatric subjects (CTRL; n = 20) and schizophrenia patients (SCZ; n = 20), as well as in the cerebellum (CB; 10 CTRL and 9 SCZ). All demographic characteristics, such as age, sex and medication of study subjects are reported in Table S1. Using high-coverage targeted bisulfite sequencing, we investigated the DNA methylation state at 10 CpG sites (−40; −30; −26; +11; +30; +74; +82; +105; +153 and +193) surrounding the *DAO* transcriptional start site (TSS) (Fig. 1A). First, we compared the methylation average at each single CpG site between CTRL and SCZ groups in each brain region (Fig. 1B). No differences between CTRL and SCZ groups were detected at each analyzed CpG sites both in HIPP and DLPFC areas. When we compared single CpG methylation state in cerebellum area, slight differences at +11, +30, +82, +105 and +153 CpG sites were found between schizophrenia-affected patients and non-psychiatric controls (Fig. 1B). However, these differences were not statistically significant after Bonferroni correction. Subsequently, we determined the average methylation at the *DAO* promoter region in CTRL and SCZ groups. We found no significant differences in any of the analyzed brain areas between the two diagnoses (Fig. 1C). In addition, we evaluated the percentage of methylation at CpH sites (CpA, CpT, CpC) in the analyzed *DAO* promoter region. We found a low degree of non-CpGs methylation in all analyzed samples. (Table S2 and Fig. 1D).

We also analyzed *DA*O mRNA expression. As previously reported^47^, our data indicated very low *DAO* mRNA expression in both HIPP and DLPFC, regardless of the diagnosis (Fig. 1E). Conversely, *DAO* mRNA levels were higher in the CB, without significant differences between CTRL and SCZ subjects (Fig. 1E).

### Single molecules *DAO* methylation analyses in non-psychiatric subjects and patients with schizophrenia

**Figure 2:**
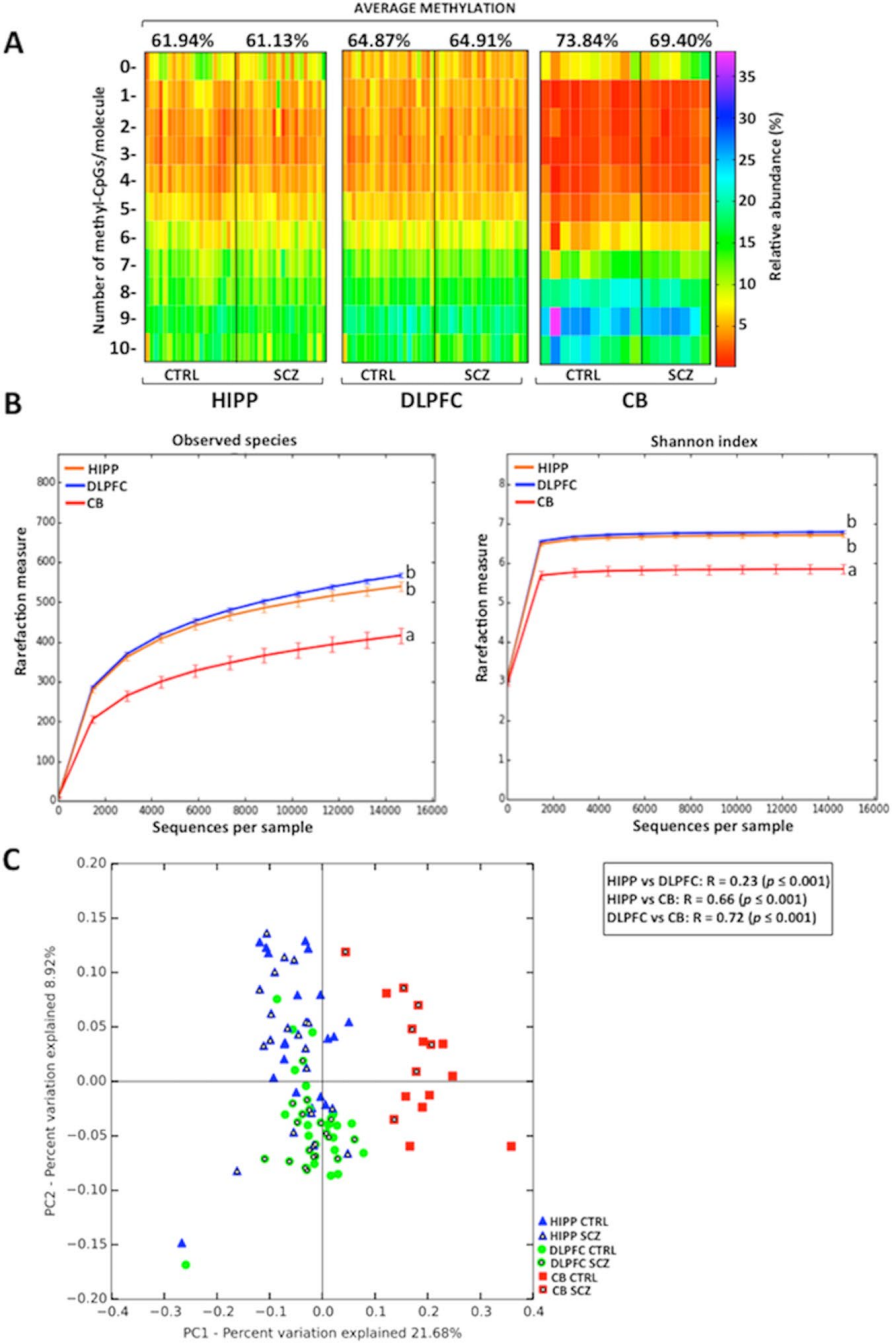
*DAO* epiallelic classes and distribution analyses. (**A)** Heatmaps show the relative abundance of different epiallelic classes (from 0 to 10 methyl-cytosine per molecule) in each sample and in all analyzed brain regions (hippocampus = HIPP; dorsolateral prefrontal cortex = DLPFC; cerebellum = CB). The color gradient from red to violet indicates the percentage of each epiallelic class. On the top of the graph, the average methylation for non-psychiatric controls (CTRL) and schizophrenia patients (SCZ) is reported for each brain area. (**B**) Epiallelic heterogeneity in each brain region has been assessed employing two different alpha-diversity metrics (number of observed epialleles and shannon index) and compared using one-way analysis of variance (ANOVA) followed by Tukey’s multiple comparison *post-hoc* test. No statistical significance was found between CTRL and SCZ groups either for observed species or for Shannon index (data not shown). Results are represented as mean ± standard error. Different letters indicate statistically significant differences among brain areas (Observed epialleles metric: HIPP vs DLPFC *p* = 0.16; HIPP vs CB *p* = 0.003; DLPFC vs CB *p* = 0.003; Shannon index: HIPP vs DLPFC *p* = 0.69; HIPP vs CB *p* = 0.003; DLPFC vs CB *p* = 0.003). (**C**) DAO epiallelic composition is reported in Bray Curtis-based 2D Principal Coordinate Analysis (PCoA) plot. Analysis of similarities (ANOSIM) with 999 permutations was used to detect the statistical significant differences in epialleles distribution among brain areas, grouping together controls and patients with schizophrenia. PC1 and PC2 in PCoA plot explained the percentage of the observed variance. On the top right, *p-values* and R are reported. No significant differences in any brain area were observed by comparing CTRL and SCZ groups (data not shown).

Based on our recent findings demonstrating the importance of methylation differences between individual cells during brain development^48–50^, we applied here an epialleles distribution analysis to evaluate *DAO* promoter methylation in CTRL and SCZ samples. The term “epiallele” is used to indicate a specific arrangement of methylated CpGs on each single *DAO* allele within the analyzed region. These analyses may be conducted either by merely considering the number of methylated CpG sites/molecule (epialleles classes analysis) or taking into account all the possible combinations of methylated CpG sites (epialleles distribution analysis). Both analyses involve the availability of a very high number of sequences per sample covering a specific region (in this study, about 200,000/loci/sample) in order to provide in depth information about DNA methylation differences between individual cells within a given population. We first performed the epialleles class analysis and plotted the results as a heatmap (Fig. 2A). No evident differences between CTRL and SCZ groups were observed. Conversely, we found striking differences among the profiles drawn for different brain areas, despite the moderate differences observed in average methylation among areas. The different frequency of specific classes accounted for the peculiar profile observed in CB, a feature that makes this brain region clearly distinguishable from HIPP and DLPFC (Figs 2A and S1; Cramer test: p < 0.001). In addition, a slight but statistically significant difference between HIPP and DLPFC (p = 0.01) was detected (see also box plot in Fig. S1). The distinctive area-specific patterns may possibly reflect the different cell type composition among the analyzed brain areas and/or the functional state of the gene in individual cells. Notably in CB, 4 out of 7 SCZ patients displayed a percentage of the un-methylated class ≥10%, while in CTRL group only 1 out of 10 subjects presented the percentage ≥10% (Fig. 2A). This may potentially reflect a greater number of *DAO* expressing cells in CB of SCZ compared to CTRL groups. Next, we performed epialleles distribution analysis. Since the *DAO* analyzed region includes 10 CpGs (Fig. 1A), the maximum number of epiallele species was 1024 (2^10^). An average of 200.000/sequences/sample were considered and analyzed through the pipeline software Amplimethprofiler^50^ (freely available at https://sourceforge.net/projects/amplimethprofiler) in order to calculate the relative frequency of all possible epialleles. To overcome biases due to a different number of sequences per sample, all the analyses were carried out upon rarefaction. We performed alpha-diversity (observed epialleles and shannon index metrics) and beta-diversity (Bray-Curtis metric) analyses to evaluate whether HIPP, DLPFC and CB were distinguishable in terms of epialleles frequency distribution. With alpha-diversity analysis, we evaluated the effective number (observed epialleles) and epialleles diversity in terms of richness and evenness (shannon index) in each sample. As shown in Fig. 2B, CB exhibited a lower epialleles species amount (ANOVA, *p* < 0.05) and a lower epiallelic diversity rate (ANOVA, *p* < 0.05) compared to HIPP and DLPFC, indicating that these last two regions seem to have more inter-cellular differences in methylation patterns. The Principal coordinate analysis (PcoA) plot (Fig. 2C), based on the epiallelic composition similarities among all individual samples, displayed a significant and striking clustering of HIPP and DLPFC against CB areas (ANOSIM R statistic, shown in the Fig. 2C).

### DNA methylation and mRNA expression of *G72* gene in non-psychiatric subjects and patients with schizophrenia

**Figure 3:**
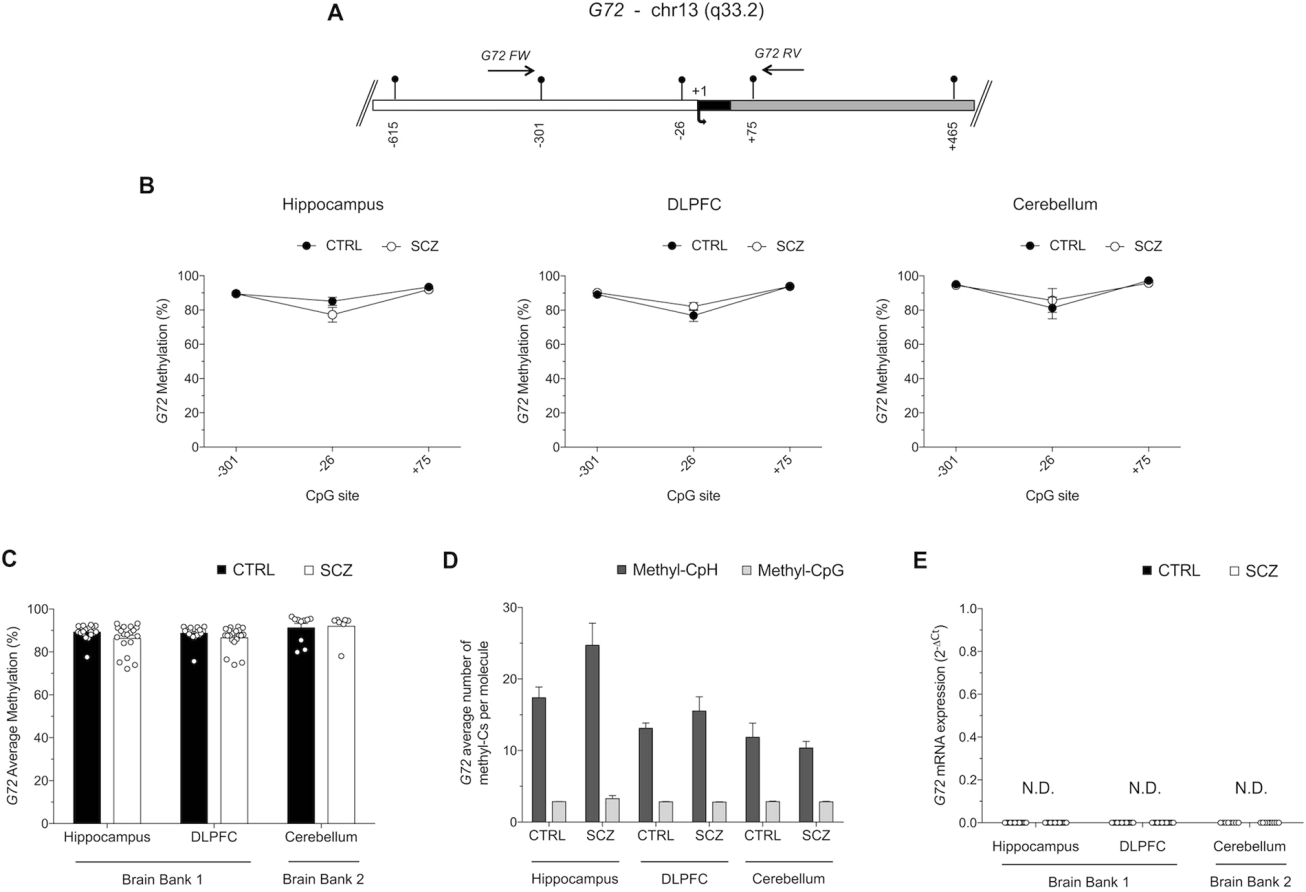
Methylation analyses at the *G72* promoter region. (**A**) *G72* putative regulatory upstream region (white box), exons (black box) and first intron (grey box) is shown. Arrow with +1 denotes the transcriptional start site. Each CpG site is indicated with vertical bars and horizontal arrows represents primers position. Gene sequence was retrieved by Ensemble database accession number: DAOA ENSG00000182346. (**B**) Methylation average at the three analyzed CpG sites is reported as comparison between CTRL and SCZ groups in all brain regions. Data are shown as means ± standard error (SEM). (**C**) Average methylation in non-psychiatric controls (CTRL; black circles) and in patients with schizophrenia (SCZ; open circles) is reported for all analyzed brain areas. (**D**) Average number of methyl-C at non-CpG sites and at CpG sites per each molecule is reported for all areas and for both groups. The percent values of non-CpG methylation is reported in Table S2. (**E**) G72 mRNA expression levels in the hippocampus, DLPFC and cerebellum of control and schizophrenia patients were not detectable (N.D.) by quantitative RT-PCR (up to 45 cycles). All data are shown as the average ± standard error of the mean (SEM).

The *G72* gene product, pLG72 (or DAOA), has been reported to modulate DAO activity in a positive^20^ or negative^32,33^ manner. So far, attempts to detect *G72* gene expression in different tissues have mostly failed and little information is available on its methylation^31,47^. Here we analyzed the methylation and expression of *G72* in SCZ and CTRL subjects. A region of 434 nucleotides of the *G72* promoter (from nucleotides −330 to +104), encompassing the TSS and containing only three CpGs (−301, −26, +75), was analyzed for DNA methylation (Fig. 3A). No significant differences were found when we compared methylation state at single CpG sites between CTRL and SCZ in all brain areas (Fig. 3B). The *G72* promoter region displayed an extremely high degree of average CpG methylation in both CTRL and SCZ groups across all analyzed brain regions. Similar to single CpGs, data analysis of average methylation indicated no statistically significant differences between diagnoses and brain areas (Fig. 3C).

Analysis of methylation at non-CpG sites strikingly showed that the average methylation values of non-CpGs ranged between approximately 10–30% (Table S2). Moreover, in the *G72* analyzed region we found a high amount of CpH methylation since we detected 71 CpH vs 3 CpG sites. In particular, the average number of methyl-CpH per molecule was about three-fold greater than that of methylated CpGs, indicating a robust contribution of non-CpG methylation to the overall methylation state of the *G72* promoter (Fig. 3D). Due to the very poor presence of CpG sites at *G72* promoter, epiallelic class and distribution analyses were not informative for this gene (data not shown). In line with the high percentage of methylation detected at all the three CpG sites and according to the high abundance of methylated non-CpG sites found within the *G72* promoter region, RT-PCR analysis revealed that *G72* mRNA was undetectable (up to 45 cycles) in each of the analyzed brain regions of CTRL and SCZ-affected subjects (Fig. 3E).

### DNA methylation analysis and mRNA expression of *SR* gene in non-psychiatric subjects and patients with schizophrenia

**Figure 4:**
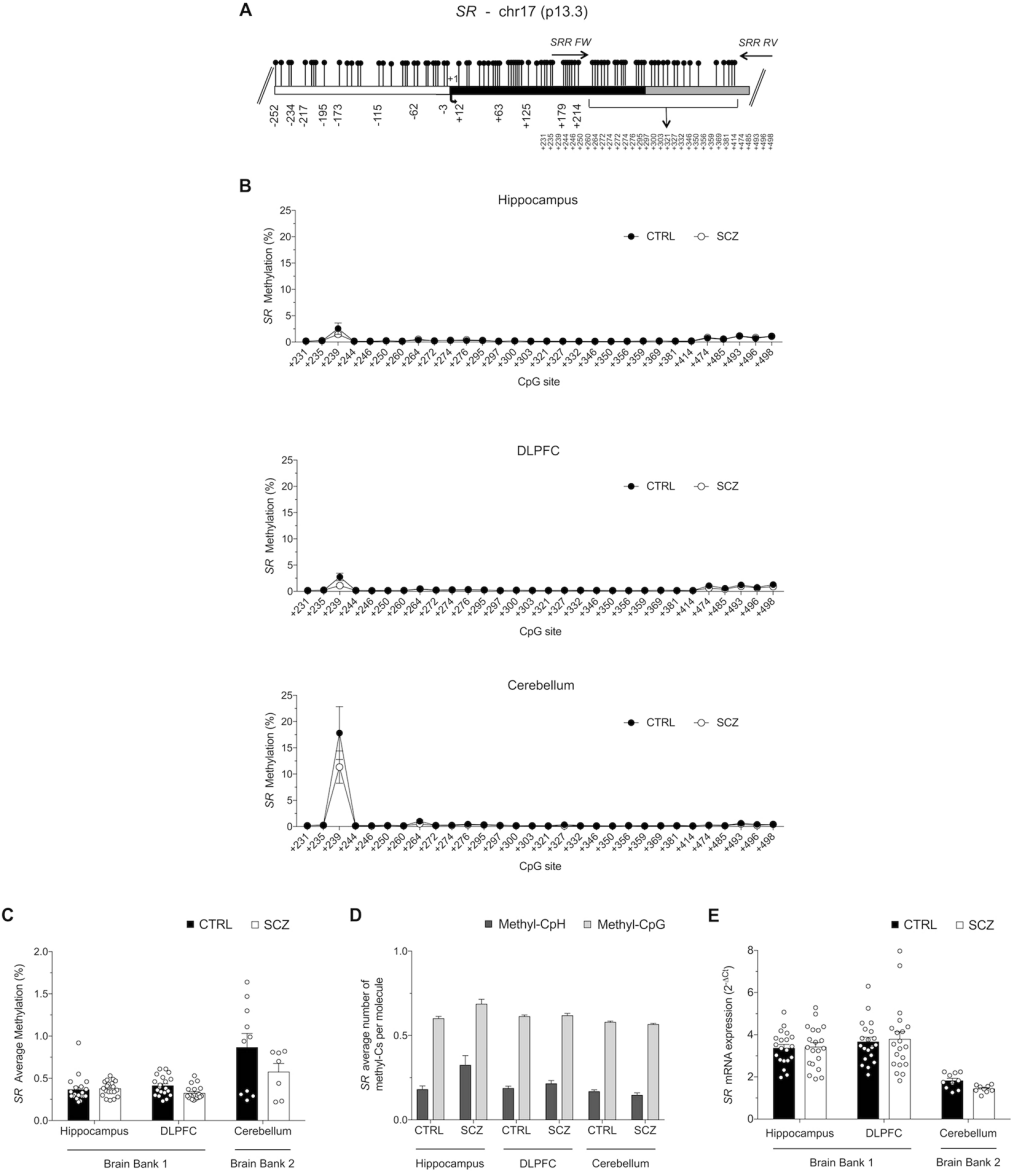
*SR* methylation and expression analyses. (**A**) CpG-rich *SR* gene structure is reported with the white box, black box and grey box representing the putative regulatory upstream region, first exon and first intron, respectively. Transcriptional start site is indicated by the black arrow. Gene sequence was retrieved by Ensemble database accession number: SRR ENSG00000167720. (**B**) Average methylation at single CpG sites for each brain region is denoted in both groups (CTRL and SCZ). (**C**) *SR* methylation levels in HIPP, DLPFC and CB areas are shown for controls and schizophrenia patients. (**D**) The level of methylation at non-CpG and CpG sites in each molecule is shown. The percent values of non-CpG methylation is reported in Table S2. (**E**) *SR* mRNA expression levels in the hippocampus, DLPFC and cerebellum of control and schizophrenia patients were detected by quantitative RT-PCR and expressed as 2^−ΔCt^. All data are shown as the average ± standard error of the mean (SEM).

The *SR* promoter is embedded in a very dense CpGs island (Fig. 4A). Attempts to analyze by bisulphite sequencing the region encompassing the TSS failed, possibly because of the very high CpG content. Thus, we shifted our analysis to the next closest region included in the same CpG island, which was located just downstream the TSS (Fig. 4A; nucleotides +209 to +522 that includes 30 CpG sites).

Overall, bisulphite sequencing showed that the whole region was practically unmethylated, irrespective of sample origin (Fig. 4B,C). In fact, a very low average methylation level, ranging from 0.23% to 1.47%, was detected in both sample groups (CTRL and SCZ) and in all analyzed brain areas (HIPP, DLPFC and CB), with no significant differences between diagnoses or brain regions (Fig. 4B,C). However, a single CpG site (+239) displayed a methylation degree far above the average, in particular in CB area (Fig. 4B). Given the extremely poor methylation level at the majority of CpG sites, analyses of epialleles classes and distribution were considered not informative for this gene (data not shown). In line with the negligible methylation levels of CpG sites of the *SR* gene region analyzed, evaluation of non-CpG methylation showed very low mCpH abundance in all brain samples (Fig. 4D and Table S2).

The very low methylation of CpG and CpH sites in the *SR* gene was mirrored by an overall appreciable *SR* mRNA expression in all brain areas analyzed (detected in the range of 30–32 RT-PCR cycles), even though no significant differences were found with diagnosis (Fig. 4E).

### DNA methylation analysis and mRNA expression of *DDO* gene in non-psychiatric subjects and patients with schizophrenia

**Figure 5:**
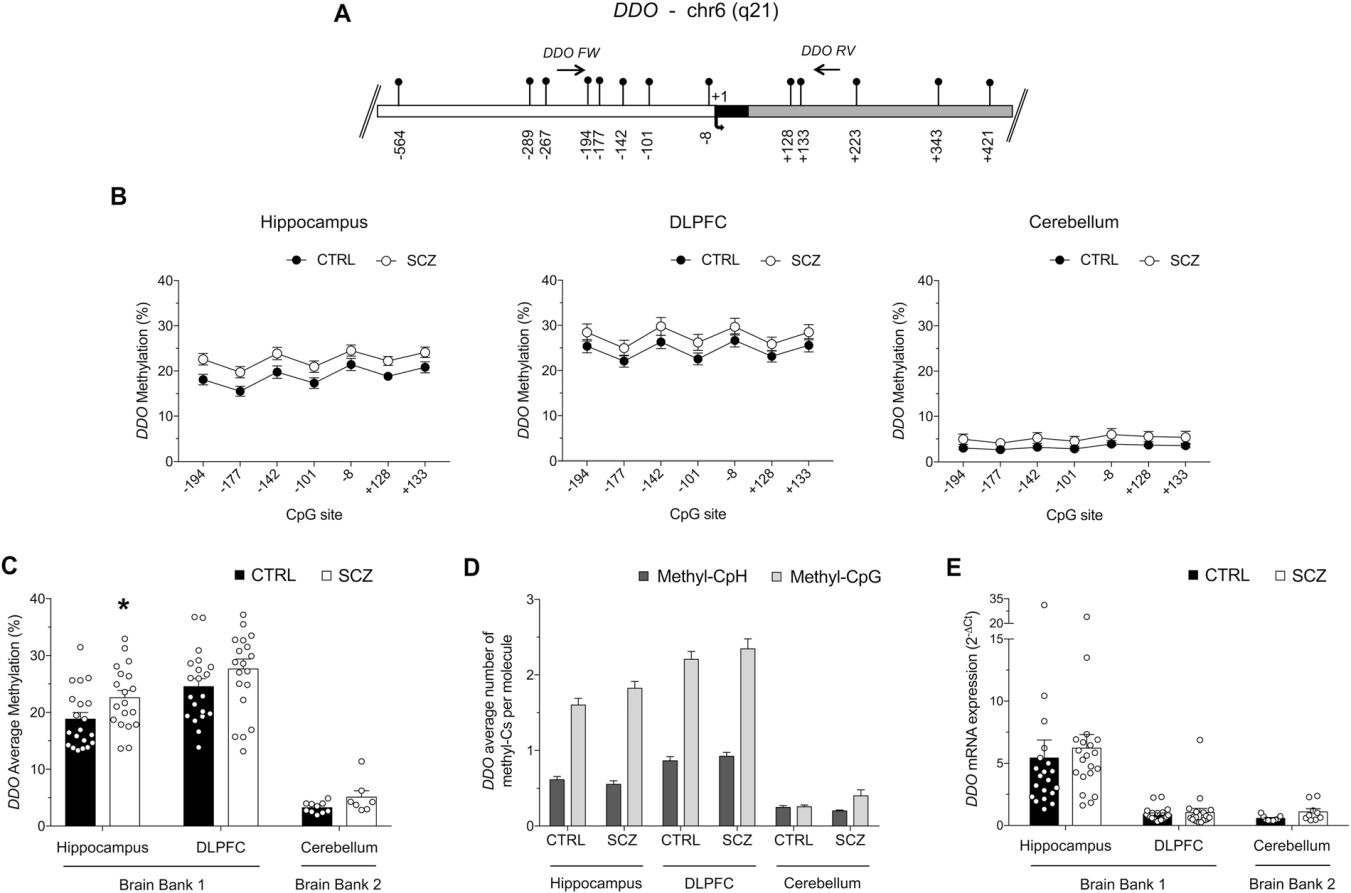
Methylation and expression studies of the *DDO* gene. (**A**) *DDO* gene structure is reported with the indication of the putative regulatory upstream region (white box), first exon (black box), first intron (grey box) and the transcriptional start site (black arrow, +1). The relative positions of each CpG site are indicated by vertical bars. Primer positions are shown as horizontal arrows. Gene sequence was retrieved by Ensemble database accession number: DDO ENSG00000203797. (**B**) Methylation levels at single CpG sites in all brain regions and for controls and patients affected by schizophrenia are reported. (**C**) DDO average methylation in CB area is shown for CTRL and SCZ groups. Data on methylation levels in HIPP and DLPFC for CTRL and SCZ subjects, previously reported in Nuzzo *et al*. 2016 (ref.^16^ in this manuscript), are here shown only in order to provide a complete overview about the DDO methylation state in different brain areas. (**D**) The average level of methylation at non-CpG and CpG sites in each molecule is shown. The percent values of non-CpG methylation is reported in Table S2. (**E**) DDO mRNA expression levels in the hippocampus, DLPFC and cerebellum of control and schizophrenia patients were detected by quantitative RT-PCR and expressed as 2^−ΔCt^. All data are shown as the average ± standard error of the mean (SEM). *Indicates *p* < 0.05.

Recently, we provided evidence for a critical role of methylation in regulating *DDO* expression^6,48^. Here we have deepened and extended the methylation and mRNA expression analysis of the *DDO* gene (see gene structure in Fig. 5A) to the cerebellum. Single CpG methylation analysis showed a moderate methylation increase in the HIPP of SCZ patients compared to CTRL subjects, in all sites analyzed except for −8 CpG site. However, after Bonferroni correction, no significant differences were found between study groups at each analyzed CpG sites (Fig. 5B). On the other hand, no significant difference between diagnoses was found in single CpGs of both DLPFC and CB (Fig. 5B). Average methylation was very low in cerebellum (about 5%) with no significant differences between schizophrenia and control groups (Fig. 5C). We found that the average *DDO* promoter methylation is significantly higher in the DLPFC than in the HIPP, both within control and schizophrenia groups. Moreover, we detected a mild but significant increase in DDO methylation levels in the HIPP of schizophrenia-affected patients, compared to control group, while no differences between diagnoses were found in the DLPFC^18^ (Fig. 5C). The levels of non-CpG methylation at the *DDO* gene were very low in CB (averages ranging from 0.25% to 0.30%) and only slightly higher in the other brain areas analyzed (Table S2 and Fig. 5D). Finally, qRT-PCR analysis showed low *DDO* mRNA expression in CB (~32 RT-PCR cycles), with no significant differences between patients and controls (Fig. 5E).

**Figure 6:**
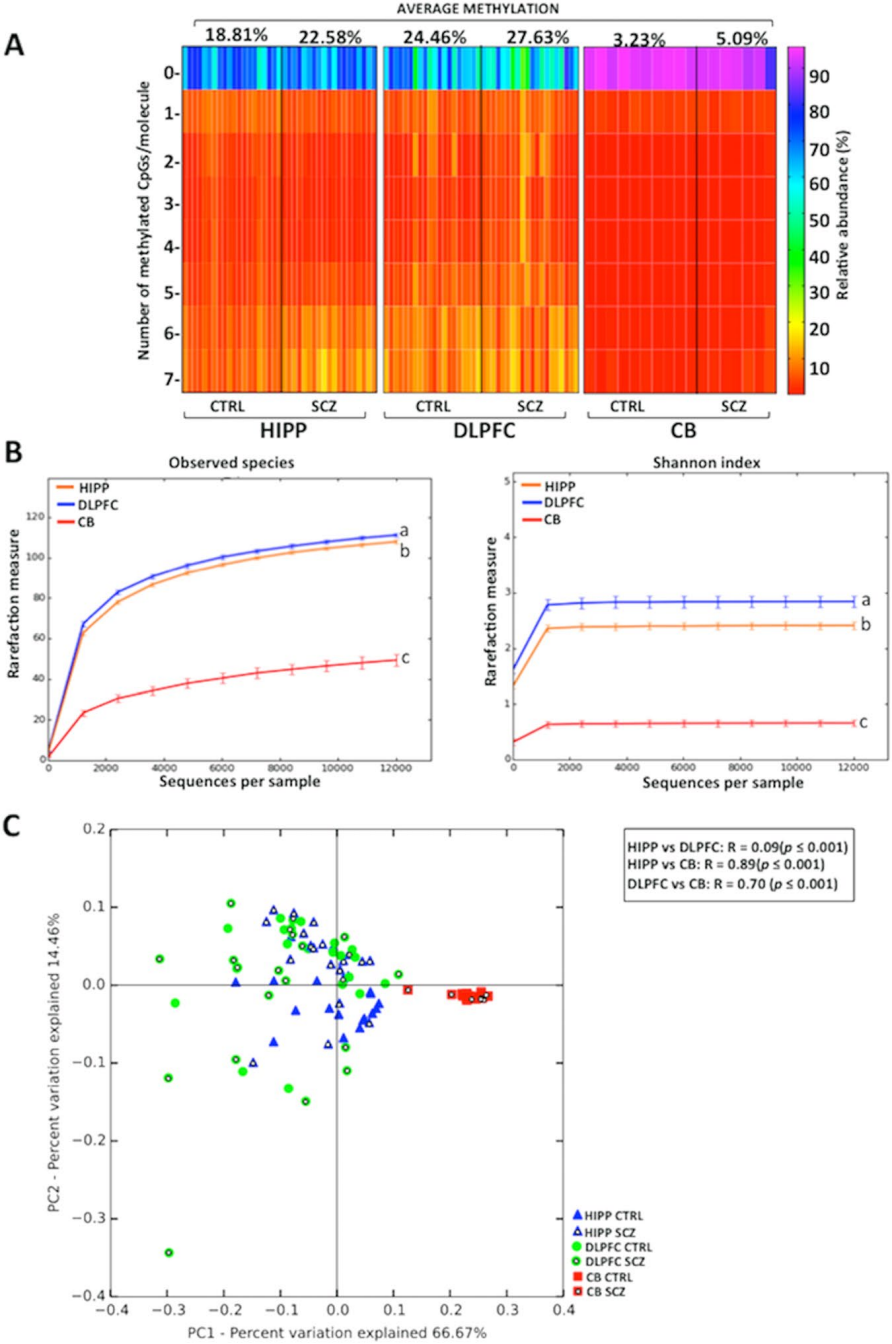
Epiallele composition analyses in *DDO*. (**A**) Heatmaps show the epiallelic classes abundance in each brain area (hippocampus = HIPP, dorso-lateral prefrontal cortex = DLPFC and cerebellum = CB) and for both groups (non-psychiatric controls = CTRL and patients with schizophrenia = SCZ). The color scale (from red to violet) indicates the frequency of each epiallelic class. On the top of the graph, the average methylation for CTRL and SCZ groups is reported for each brain area. (**B**) Two alpha diversity metrics, observed epialleles and Shannon index, are reported. Statistical differences, indicated by different letters, were assessed using one-way analysis of variance (ANOVA) followed by Tukey’s multiple comparison *post-hoc* test (Observed epialleles metric: HIPP vs DLPFC *p* = 0.02; HIPP vs CB *p* = 0.003; DLPFC vs CB *p* = 0.003; Shannon index metric: HIPP vs DLPFC *p* = 0.006; HIPP vs CB *p* = 0.003; DLPFC vs CB *p* = 0.003;). No statistically significant differences were observed between CTRL and SCZ groups (data not shown). Results are represented as mean ± standard error of the mean (SEM). (**C**) DDO epiallelic composition is reported in Bray Curtis-based 2D Principal Coordinate Analysis (PCoA) plot. Analysis of similarities (ANOSIM) with 999 permutations was used to detect the statistical significant differences in epialleles distribution among brain areas, grouping together controls and patients with schizophrenia. PC1 and PC2 in PCoA plot explained the percentage of the observed variance. On the top right, *p-values* and R are reported. No significant differences in any brain area were observed by comparing CTRL and SCZ groups (data not shown).

As expected by the lower average methylation, when we performed epialleles classes analysis we found that cerebellum displayed a clearly different pattern compared to HIPP and DLPFC (Figs 6A and S2), as confirmed by the Cramer test (p < 0.001). However, although sharing a similar average methylation, DLPFC and hippocampus displayed significant differences in epialleles classes distribution (Figs 6A and S2), as detected by the Cramer test (p = 0.03). Furthermore, alpha diversity metrics displayed significant differences (ANOVA, p < 0.05) in the number of observed epialleles and shannon index among HIPP, DLPFC and CB areas (Fig. 6B). Also in this case, the cerebellum seems to show a lower level of inter-cellular methylation differences. Finally, the PcoA plot confirmed that the CB clearly differed in terms of epialleles distribution from HIPP and DLPFC indicating that epiallelic patterns strongly typified CB cells with respect to the other two areas (Fig. 6C). Although we did not detect schizophrenia-related patterns at the investigated loci, the ability to detect brain region specific profiles in terms of individual cell methylation patterns, demonstrates the feasibility for an epiallelic approach to distinguish disease-related profiles at relevant loci in psychiatric conditions.

## Discussion

D-Ser and D-Asp act, respectively, as a co-agonist and agonist of NMDARs. Based on their modulatory role on NMDAR-mediated neurotransmission, converging evidence over recent years has implicated D-Ser and D-Asp in the pathophysiology of schizophrenia. In line with this, genetic studies have also shown that different SNPs within *SR*, *DAO* and *G72* are associated with schizophrenia^20,51–53^. Moreover, a genome-wide association study of up to 36,989 patients and 113,075 controls identified SR among the 108 loci strongly associated to this psychiatric disease^24^. To date, however, very few studies^18,47^ have addressed DNA methylation status and mRNA expression of human D-Ser and D-Asp regulating genes. In light of this gap in knowledge, we provide for the first time a comprehensive representation of CpG and CpH methylation status and mRNA expression levels of *DAO*, *G72*, *SR* and *DDO* genes in three different post-mortem brain areas of patients with schizophrenia and non-psychiatric controls from two different brain banks by a novel, ultradeep approach^48–50,54,55^. Interestingly, despite the lack of main alterations between diagnosis groups, we were able to uncover clear differences among the HIPP, DLPFC and CB, with sharply recognizable methylation signatures for most of the analyzed genes.

Among the genes analyzed in this study, the CpG methylation analysis of *DAO* best exemplifies the power of the proposed approach, based on the examination of cell to cell methylation differences. The average methylation values, either measured at individual sites or integrating all the 10 CpG sites contained in the analyzed region, showed a high methylation degree in all areas. These data are consistent with very low *DAO* expression in the HIPP and DLPFC. However, we found neither significant differences among DLPFC, HIPP and CB nor association with schizophrenia. Interestingly, the *DAO* gene promoter displayed a CpG richness (10 CpG in a 325 bp region) and methylation degree (60–70%) that allowed us to perform further analyses based on the evaluation of methylated CpG combinations at single allele level. Consistently, we detected brain region-specific epiallelic profiles in patients and non-psychiatric controls. These sets of peculiar methylation signatures, regardless of diagnosis, render the CB clearly distinguishable from DLPFC and HIPP, and possibly reflect differences in: cellular composition, epialleles distribution among cells, and a specific CpG combinatorial code. Although DLPFC and HIPP showed similarly low mRNA levels and shared similar *DAO* epiallelic distribution, we were able to detect few, but clearly distinctive epiallelic signatures (Figs 2 and S1). These included a depletion of low-intermediate epiallelic classes and a relative poorness of epiallelic species (measured by alpha-diversity) in CB (Fig. 2B). In addition, a clear clustering of cerebellum-derived specimens was observed by qualitative analyses of epiallelic configurations in individual samples (PCoA plot in Fig. 2C). These different signatures may reflect fine differences in cell populations containing the *DAO* gene in a specific functional state. By this point of view, we believe that our approach represents a novel and powerful tool to detect fine differences in gene methylation distribution, otherwise not appreciated with classical targeted or genome-wide methods. On the other hand, the same analysis applied to *G72* and *SR* was less informative, due to the CpG architecture and methylation state of these genes.

*G72* was heavily, almost fully, methylated in the few CpG sites present at the promoter region and showed a high degree of CpH methylation in all analyzed samples, regardless of brain region and diagnosis, thus possibly explaining the ubiquitously repressed state of the gene, also reported by others^47^. The existence and the functional role of non CpG methylation in the brain has been recently investigated^44,46,56,57^. Consistent with our findings, it has been found that enriched CpH methylation at promoter regions is associated with gene repression, possibly mediated by MeCP2 binding^57^. The very high degree of non-CpG methylation (ranging from about 15 to 35%) at the *G72* promoter is even higher than mean global levels reported in the adult brain (about 10%)^46^. Despite the methodological difficulties in measuring non-CpG methylation due to possible incomplete bisulfite conversion, the following factors make us confident about the genuine nature of the detected CpH methylation: (1) the use of unmethylated spike-in controls, (2) the homogeneity of profiles observed within individual groups and technical replicates, and (3) the much higher non-CpG methylation degree consistently observed at *G72*, compared to *DAO*, *SR* and *DDO*.

To our knowledge, no studies have investigated the methylation state of *SR*, which was recently identified as a risk gene for schizophrenia^24^. Interestingly, we show that *SR* is embedded in a very dense CpG island, which was almost totally unmethylated in all study samples, possibly explaining the active state of the *SR* gene. We detected a higher level of methylation in the CB, as compared to HP and DLPFC, which is consistent with the lower levels of SR in adulthood in CB. Consistently, we found a very low degree of non-CpG methylation at *SR* regulatory region. However, we did not find changes in either *SR* methylation or mRNA expression levels across all analyzed brain regions between patients with SCZ and controls.

Finally, we extended to the CB of subjects with or without SCZ diagnosis the methylation and expression analysis of *DDO*, previously performed only as average values in the hippocampus and DLPFC^18^. We reported that *DDO* was less methylated and more expressed in the DLPFC compared to the HIPP, regardless of the diagnosis^18^. Here we found that both *DDO* methylation (at both CpG and non CpG sites) and expression were lower in the CB compared to the HIPP and DLPFC. This finding is consistent with mechanisms other than DNA methylation in controlling *DDO* gene activity in the cerebellum.

The lack of significant methylation and expression changes in *DAO*, *G72*, *SR* and *DDO* genes between patients with schizophrenia and non-psychiatric controls could be due to several reasons. First, schizophrenia has a multifactorial etiology, with multiple genetic insults with small-effects interacting with the environment to produce a heterogeneous disorder. In this regard, although multiple independent studies have found reduced SR protein expression and D-serine in schizophrenia, other studies have found SR levels to be increased or unaltered in the brains of patients with schizophrenia^13,15,18,21,22,51,58,59^. Moreover, given the small number of samples analyzed here, it is possible that an increased sample size is necessary to detect more subtle changes in gene expression. These considerations have limited significance for *G72* and *DAO* since the former gene is highly methylated and essentially unexpressed in all the brain regions analyzed, while the latter shows a high degree of methylation and very low expression levels in the hippocampus and DLPFC, two brain regions prominently affected in schizophrenia. However, based on their significant expression in the cerebellum, we cannot exclude that changes in *DAO*, as well as *SR* and *DDO* expression, could contribute to the pathophysiology of SCZ; although there is no clear consensus about a cerebellar involvement in schizophrenia^60^. Another critical point to take into account is the effect of potential confounding variables, such as *post-mortem* interval, age, and pH of the brain samples. However, statistical analyses showed that confounders did not affect the RNA expression and DNA methylation results (see Materials and Methods).

Importantly, while the methylation states of *SR* (very low) and *G72* (very high) comports with the overall high and low levels of expression of these genes, respectively, additional epigenetic mechanisms likely contribute to the differences between methylation and expression levels in *DAO* and *DDO*. While *DAO* shows a high degree of methylation in all brain regions, it is significantly expressed only in the cerebellum. Although *DDO* methylation levels are comparable between hippocampus and DLPFC and much lower in the cerebellum, *DDO* gene expression is high in the hippocampus and much lower in the DLPFC and cerebellum. Thus it is possible that epigenetic mechanisms, other than DNA methylation, such as chromatin remodeling or miRNA expression, may underlie the observed phenomena. Besides epigenetic regulation, we cannot exclude the possibility that translational mechanisms may also regulate the expression of SR, DAO, G72 and DDO proteins, thus ultimately affecting D-Ser and D-Asp metabolism.

In conclusion, epiallele classes and configuration analyses were able to provide distinct area-specific patterns suggesting the occurrence of an orchestrated distribution of epialleles in diverse cell populations represented in each brain area. As it is possible that the methylation state of the genes interrogated here varies between different cell types (i.e. excitatory and inhibitory neurons, astrocytes), there could be differences in SCZ if these diverse cell populations could be sorted prior to sequencing. Furthermore, differences in DNA methylation and gene expression in SCZ could emerge if there was more precise anatomical sampling, that is DNA isolation from specific cortical layers or hippocampal subfields. Overall, we believe that at selected genes, epiallele-based analyses may be more informative than traditional methylation analyses and can be successfully utilized in a broad range of applications, including in depth determination of epigenetic origin of brain diseases. In addition, provided that these analyses are successfully transferable to peripheral cells, they may be applied for diagnostic and clinical purposes.

## Methods

### Human Tissue Samples Collection

Dorsolateral prefrontal cortex and hippocampal samples from post-mortem brains were obtained from The Human Brain and Spinal Fluid Resource Center, Los Angeles Healthcare Center, Los Angeles, CA, USA (Brain Bank 1, BB1). Cerebellum tissue samples were obtained from the MRC London Neurodegenerative Disease Brain Bank of the Institute of Psychiatry, King’s College London, UK (Brain Bank 2, BB2). All tissues were carried out under the regulations and licenses of the Human Tissue Authority and in accordance with the Human Tissue Act of 2004. Clinical diagnosis of SCZ was performed according to DSMIII-R criteria. Frozen tissues were pulverized in liquid nitrogen and stored at −80 °C for subsequent processing. A detailed description of all the samples is shown in Table S1.

### DNA extraction and methylation analysis

DNA was extracted from a portion of liquid nitrogen pulverized post-mortem brain tissues, using Dneasy Blood & Tissue Kit (Qiagen, Hilden, Germany), following the manufacturer’s instructions. DNA was quality checked using NanoDrop 2000, (Thermo Scientific) and quantified using Qubit 2.0 Fluorometer (Invitrogen). 1 gamma of genomic DNA was converted by sodium bisulfite with EZ DNA Methylation Kit (Zymo Research) according to the manufacturer’s instruction. A first PCR step was performed using bisulfite-specific primers described in Table 1. Reactions were performed in 30 µl total volumes: 3 µl 10x reaction buffer, 0.6 µl of 10 mM dNTP mix, 0.9 µl of 5 mM forward and reverse primers, 3.6 µl MgCl2 25 mM, 2–4 µl bisulfite template DNA, 0.25 µl FastStart Taq, and H2O up to the final volume. Sequences of gene-specific primers, along with individual PCR conditions, are reported in Table 1. The 5′ end of these primers contains overhang adaptor sequences (FW: 5′ TCGTCGGCAGCGTCAGATGTGTATAAGAGACAG ‘3; RV: 5′ GTCTCGTGGGCTCGGAGATGTGTATAAGAGACAG ‘3) that were used in a second step of PCR to add multiplexing indices and Illumina sequencing adaptors. Second PCR step was performed in 50 µl total volumes: 5 µl 10x reaction buffer, 1 µl dNTP mix, 5 µl forward and reverse “Nextera XT” primers (Illumina, San Diego, CA), 6 µl 25 mM MgCl2, 5 µl of first PCR product, 0.4 µl FastStart Taq, and H_2_O up to the final volume.

**Table 1:** Primers and amplification conditions used in the first PCR step for all genes in this study.

GENE	AMPLICONS	FW PRIMER	RV PRIMER	Amplification conditions
*DAO*	−104/+221	aaggTTtgtTTaTaggggTttgaga	ccaActcaaaAAtAcatctAccactc	Denature at 95 °C for 2 min; 35 cycles of denaturing at 95 °C for 30 s, annealing at 54 °C for 40 s, and extension at 72 °C for 50 s. Final elongation at 72 °C for 6 m
*G72*	−330/+104	aaTaatattgatTttgagttaatt	ccatAcaaatAtcacattAcact	Denature at 95 °C for 2 min; 38 cycles of denaturing at 95 °C for 30 s, annealing at 57 °C for 40 s, and extension at 72 °C for 50 s. Final elongation at 72 °C for 6 m
*SR*	+209/+522	GgagYGagagTTtgggtggg	cttcaaAAtcccaaAtaatAAc	Denature at 95 °C for 2 min; 35 cycles of denaturing at 95 °C for 30 s, annealing at 57 °C for 40 s, and extension at 72 °C for 50 s. Final elongation at 72 °C for 6 m
*DDO*	−227/+171	aTTtaTaaatTagTtggagaaagTTTag	cctattcaAacacactcccaaactcc	Denature at 95 °C for 2 min; 36 cycles of denaturing at 95 °C for 30 s, annealing at 52 °C for 40 s, and extension at 72 °C for 50 s. Final elongation at 72 °C for 6 m
*M13mp18*	5946/6294	Ggtgaagggtaattagttgttgtt	ccaataccaaacttacatacct	Denature at 95 °C for 2 min; 33 cycles of denaturing at 95 °C for 30 s, annealing at 57 °C for 40 s, and extension at 72 °C for 50 s. Final elongation at 72 °C for 6 m

The capital letters in the primers sequences indicate the original C or G. For all genes, the positions refer to TSS; for M13mp18 the position refers to sequence entry X02513.1 (NCBI, GenBank).

To eliminate primers dimers, a purification phase after both PCR steps was performed using AMPure purification magnetic Beads (Beckman-Coulter, Brea, CA) following the manufacturer’s protocol. All amplicons were quantified using Qubit^®^ 2.0 Fluorometer. A library of bisulfite treated amplicons was obtained pooling amplicons at equimolar ratio and then diluted to final concentration of 8 picomolar. Phix control libraries (Illumina) were combined with normalized library [10% (v/v)] to increase diversity of base calling during sequencing. Amplicons library was subjected to sequencing using V3 reagents kits on Illumina MiSeq system (Illumina). Paired-end sequencing was performed in 281 cycles per read (281 × 2). An average of 200,000 reads/sample were obtained. To estimate the rate of bisulfite conversion, fully unmethylated M13mp18 double strand DNA (New England BioLabs) was added in 10 representative samples.

### mRNA extraction and RT PCR analysis

Total RNA was extracted using miRNeasy® kit (Qiagen, Hilden, Germany) according to the manufacturer’s instructions. Total RNA (0.5 μg per sample) was used to synthesize cDNA, using Qiagen QuantiTect Reverse Transcription kit (Qiagen, Hilden, Germany).

*DDO*, *DAO* and *SR* mRNA expression levels were measured by quantitative RT-PCR with Real Time Ready Assays (Roche Diagnostics) and LightCycler® 480 Probe Master (Roche Diagnostics) using a Light Cycler 480 Real Time PCR system with 96-well format (Roche Diagnostics). The following amplification protocol (Monocolor Hydrolysis PROBE program) was used: 10 s for initial denaturation at 95 °C followed by 45 cycles consisting of 10 s at 95 °C for denaturation, 30 s at 60 °C for annealing, and 1 s for elongation at 72 °C temperature. The following probes (Roche Diagnostics) were used for cDNA amplification of *DDO*, *DAO*, *SR* and of the housekeeping genes, β-actin and cyclophilin (PP1A): DDO, Fw: GGTGTTCATTTGGTATCAGGTTG, Rev: CTTTCGAAATCCCAGAACCA (catalog assay number 2727); DAO, Fw: GGCATCTACAATTCCCCGTA, Rev: TGGAAGATGCCTCCAAGAGT (Catalog assay number 113968); SR, Fw: GGAATGCTTGCTGGAATAGC, Rev: GCAGTCATCTGCATTTGAG (Catalog assay number 117079); β-actin, Fw: TCCTCCCTGGAGAAGAGCTA; Rev: CGTGGATGCCACAGGACT (Catalog assay number 132); PP1A, Fw: TTCATCTGCACTGCCAAGAC; Rev: CACTTTGCCAAACACCACAT (Catalog assay number 9253). *G72* mRNA expression levels were detected by using LightCycler 480 SYBR Green I Master (Roche Diagnostic) in a LightCycler 480 Real Time thermocycler (Roche). The following protocol was used: 10 s for initial denaturation at 95 °C followed by 45 cycles consisting of 10 s at 94 °C for denaturation, 10 s at 60 °C for annealing, and 6 s for elongation at 72 °C temperature. The primers used for G72 cDNA amplification were the following: G72 forward: 5′-tgggtgctgactccag-3′, G72 reverse: 5′-gttaccgtctttcttc-3′. Primers used for the amplification of the housekeeping genes, β-actin and PP1A, were: β-actin forward: 5′-catccgcaaagacctgtacg-3′, β-actin reverse: 5′-cctgcttgctgatccacatc-3′; PP1A forward: 5′-ggaggctttgaggttttgca-3′, PP1A reverse: 5′-ttaaggtgggcagagaaggg-3′. All measurements from each subject were performed in duplicate. Expression levels of all the transcripts analyzed were normalized to the geometric mean of the two housekeeping genes, β-actin and PP1A, and expressed as 2^−ΔCt^.

### Bioinformatics and statistical analyses

FASTQ paired-end reads obtained (Illumina MiSeq) were assembled using PEAR^61^ tool with a minimum overlapping regions of at least 40 residues. Only sequences with an average PHRED score ≥ 33 were quality filtered and converted in FASTA format using PRINSEQ^62^ tool. Methylation analyses of each passing filter amplicon were conducted using the pipeline software “Amplimethprofiler” (https://sourceforge.net/projects/amplimethprofiler/), specifically designed for handling epialleles analyses^50^. All sequences were filtered at a specific threshold for: expected length of reads, bisulfite efficiency, maximum percentage of ambiguously aligned CG sites and percent of aligned read, generating a quality filtered FASTA file. Using BLASTn, filtered sequences were then aligned to the corresponding bisulfite converted reference. For each sample, a summary with information about the number of reads passing filters, the methylation status at each CpG site, and the bisulfite efficiency (spiked-in) was generated. Moreover, a methylation profiles matrix was obtained for each molecule assigning 0, 1 and 2 values to each CpG dinucleotide if the site was recognized as unmethylated, methylated or ambiguous, respectively. Considering each row of the matrix as an epiallele, the pipeline generated a tabular format file (BIOM format) containing methylation profiles count for all samples. The BIOM table was processed with QIIME scripts^63^ to obtain alpha and beta diversity measures. To avoid biases due to different number of sequences/sample, all the subsequent analyses were conducted on the same number of reads normalizing samples with a rarefaction procedure^63^. Epiallelic classes analysis was obtained from a rarefied BIOM table considering the number of methyl-cytosines per molecule independent of their position. To assess epiallelic heterogeneity within each sample, two alpha diversity metrics, number of observed epialleles and shannon index, were employed and compared using one-way Analysis of Variance (ANOVA) followed by Tukey’s multiple comparison *post-hoc* test. Bray Curtis dissimilarity was used to evaluate the epiallelic distribution differences among all samples (beta diversity). To test the force of brain area grouping in terms of epialleles structure, Analysis of Similarities (ANOSIM) method with 999 permutations was used. Methylation average at non-CpG sites (mCpH) was analyzed using the filtered FASTA format file for each sample. Number of CpA, CpC and CpT was calculated in each amplicon for all different genes and indicated as “total CpH”. Number of methylated-cytosine at non-CpG sites was counted for each sample and indicated as the “amount of mCpH”. To obtain the percentage of mCpH in all samples, we then applied the following formula: (amount of mCpH/total CpH) × 100. Methyl CpH per molecule was calculated as follows: amount of mCpH/number of reads. Percentage of C found in spike-in controls was subtracted in order to obtain the presumed average number of methylated CpH per molecule.

Methylation average data are expressed as means ± standard error (SEM). Comparisons between groups were performed using Student’s t test with α significance level <0.05. Comparisons between methylation classes profiles were assessed by the two-sample Cramer-Test^64^ using the package “cramer” in the R statistical environment^65^. This nonparametric two-sample-test on equality of the underlying distributions is a suitable method for this kind of multivariate analysis.

The effect of potential confounding variables, such as *post-mortem* interval, age, and pH of the brain samples, was investigated. In Brain Bank 2 patients and control were substantially balanced for the considered variables. SCZ patients in Brain Bank 1 in average were younger and had a slight longer PMI with respect to non-psychiatric subjects (Table S1, Mann-Whitney U-test, p < 0.001 and p < 0.05 respectively). Therefore, we performed also multivariable analyses adjusting the comparisons for these two potential confounders – using ANCOVA models – finding that our results were not affected by these confounders.

All statistical analyses were performed using GraphPad (GraphPad Prism Software, Inc., La Jolla, CA, USA www.graphpad.com/guides/prism/7/statistics/index.htm) and the computing environment R^65^ (http://www.R-project.org).

### Data availability

The datasets generated during the current study are available in the European Nucleotide Archive (ENA) repository, accession no PRJEB24382.

## Electronic supplementary material

Supplementary Information

Supplementary Table S1
