# Supplementary material for: DNA methylation landscape of the genes regulating D-serine and D-aspartate metabolism in post-mortem brain from controls and subjects with schizophrenia
Source: Sci Rep. 2018 Jul 5;8:10163. doi: 10.1038/s41598-018-28332-x (PMC6033866; doi:10.1038/s41598-018-28332-x)
Supplement: Supplementary file 1 — Supplementary Information [file 41598_2018_28332_MOESM1_ESM.pdf]

Supplementary Information

DNA methylation landscape of the genes regulating D-serine and D-aspartate metabolism in post-mortem brain from controls and subjects with schizophrenia

Simona Keller[1,2,+], Daniela Punzo[3,4,+], Mariella Cuomo[1,+], Ornella Affinito,[1,2] Lorena Coretti[1,2], Silvia Sacchi[5,6], Ermanno Florio[1,2,7], Francesca Lembo[1], Massimo Carella[8], Massimiliano Copetti[9], Sergio Cocozza[1,2], Darrick T. Balu[10,11], Francesco Errico[3,12], Alessandro Usiello[3,4,8,*] and Lorenzo Chiariotti[1,2,*]

[1] Department of Molecular Medicine and Medical Biotechnology, University of Naples "Federico II", 80131 Naples, Italy;
[2] Endocrinology and Molecular Oncology Institute (I.E.O.S.), National Research Council (C.N.R.), 80131 Naples, Italy;
[3] Laboratory of Behavioural Neuroscience, Ceinge Biotecnologie Avanzate, 80145, Naples, Italy;
[4] Department of Environmental, Biological and Pharmaceutical Sciences and Technologies, Università degli Studi della Campania "Luigi Vanvitelli", 81100, Caserta, Italy;
[5] Dipartimento di Biotecnologie e Scienze della Vita, Università degli Studi dell'Insubria, 21100, Varese, Italy;
[6] The Protein Factory, Politecnico di Milano and Università degli studi dell'Insubria, 20131, Milano, Italy;
[7] Department of Medicine, University of California, San Diego UCSD, La Jolla 95000 Gilman Dr.
[8] Medical Genetics Unit, IRCCS Casa Sollievo della Sofferenza, 71013, San Giovanni Rotondo, FG, Italy
[9] Unit of Biostatistics, IRCCS Casa Sollievo della Sofferenza, 71013, San Giovanni Rotondo, FG, Italy;
[10] Department of Psychiatry, Harvard Medical School, Boston, 02115, MA, USA;
[11] Translational Psychiatry Laboratory, McLean Hospital, Belmont, 02478, MA, USA;
[12] Department of Agricultural Sciences, University of Naples "Federico II", 80055, Portici, Italy.
*corresponding authors

Supplementary Figures

Figure S1

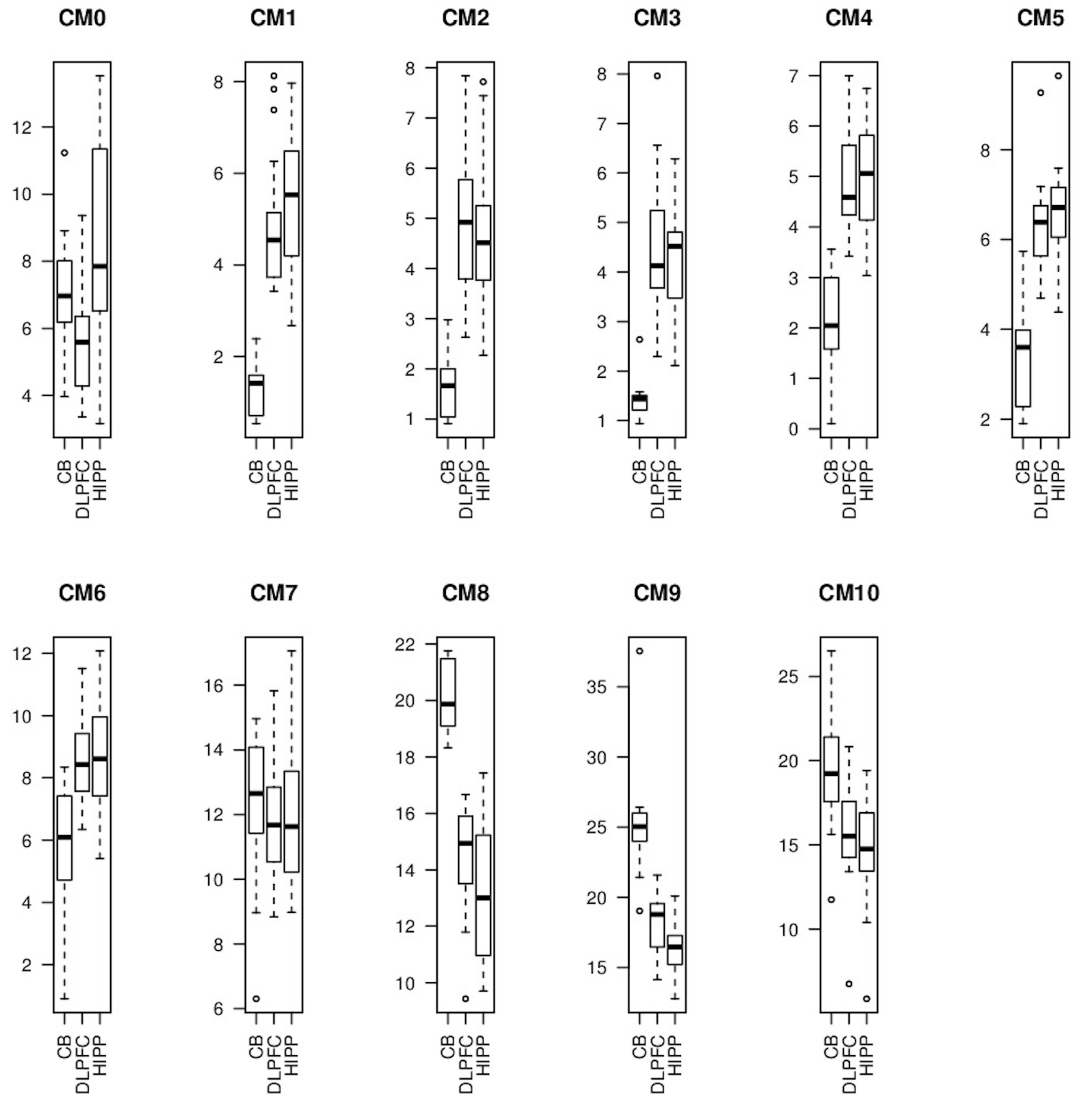

Figure S1. Distribution of the DAO epiallelic classes frequencies in the three analyzed brain regions for CTRL samples. Each boxplot represents a methylation class from unmethylated (CM0) to fully methylated (CM10). The black central line indicates the median of the distribution. White circles represent samples with an outlier value. Statistical significant differences among areas were assessed by Cramer test: HIPP vs CB $p < 0.001$; DLPFC vs CB $p < 0.001$; HIPP vs DLPFC $p = 0.03$.

Figure S2

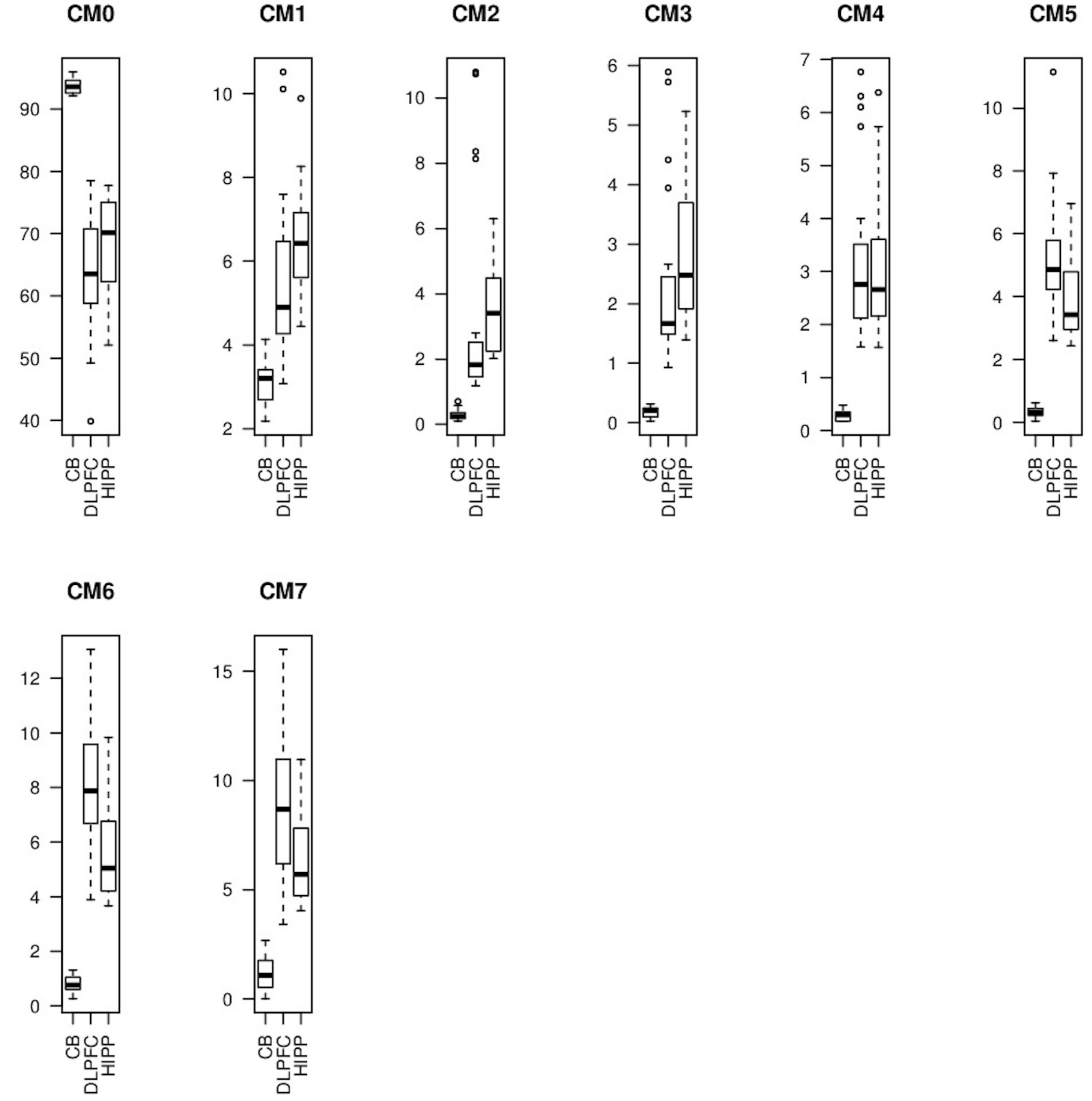

Figure S2. Distribution of the DDO epiallelic classes frequencies in the three analyzed brain regions for CTRL samples. Each boxplot represents a methylation class from unmethylated (CM0) to fully methylated (CM7). The black central line indicates the median of the distribution. White circles represent samples with an outlier value. Statistical significant differences among areas were assessed by Cramer test: HIPP vs CB $p < 0.001$; DLPFC vs CB $p < 0.001$; HIPP vs DLPFC $p = 0.01$).

Table S2

	Hippocampus		Dorso-lateral prefrontal cortex		Cerebellum	
	Non-psychiatric controls	Schizophrenia patients	Non-psychiatric controls	Schizophrenia patients	Non-psychiatric controls	Schizophrenia patients
DAO	1.12 ± 0.04	1.66 ± 0.17	1.71 ± 0.05	1.73 ± 0.10	1.25 ± 0.03	1.31 ± 0.05
DAOA	24.47 ± 2.11	34.81 ± 4.23	18.48 ± 1.03	21.87 ± 2.82	16.70 ± 2.77	14.6 ± 1.28
SRR	0.29 ± 0.04	0.53 ± 0.09	0.31 ± 0.02	0.35 ± 0.30	0.27 ± 0.02	0.24 ± 0.02
DDO	0.77 ± 0.06	0.69 ± 0.06	1.08 ± 0.07	1.15 ± 0.06	0.30 ± 0.03	0.25 ± 0.01

Table S2. Average methylation at non-CpG sites. For all genes considered in our analysis, average of mCpH for both CTRL and SCZ groups and in each brain region was assessed and indicated as mean ± SEM. Percentage of average methylation at non-CpG sites was calculated in each sample as (amount of mCpH / total number of CpH sites)*100.
